# Risk factors for catheter-related bloodstream infections in a high-risk cancer patient population

**DOI:** 10.1017/ice.2025.45

**Published:** 2025-07

**Authors:** Andrea Haddad, Rita Wilson Dib, Anne-Marie Chaftari, Ying Jiang, Mohamed Moussa, Hiba Dagher, Ann Philip, Ray Hachem, Issam Raad

**Affiliations:** Department of Infectious Diseases, Infection Control and Employee Health, Unit 1460, The University of Texas MD Anderson Cancer Center, Houston, TX, USA

## Abstract

To identify risk factors for catheter-related bloodstream infections (CRBSI) in cancer patients, we compared 200 CRBSI cases to 400 controls. Neutropenia, transplants, multiple catheters, blood products, and basilic/cephalic PICCs increased CRBSI risk, while jugular insertion was protective. Catheter site selection can reduce risk. Other targeted strategies are warranted.

## Introduction

**C**entral venous catheters (CVCs) have become essential in medicine, particularly for cancer patients requiring long-term intravenous therapies like chemotherapy, antibiotics, and blood transfusions. While improving quality of life by reducing repeated venipunctures, long-term central venous catheters (LCVCs), implanted beyond 14 days, carry risks, notably catheter-related bloodstream infections (CRBSIs), a leading cause of morbidity and mortality despite preventive measures.

This study focuses on identifying cancer patient subgroups most susceptible to CRBSI, using stringent Infectious Diseases Society of America (IDSA) criteria^1^ to differentiate true CRBSI from the broader CDC surveillance definition of central-line associated bloodstream infections (CLABSI),^2^ which may include mucosal barrier injury (MBI) infections originating from the gut rather than the catheter.

## Methods

This retrospective case-control study was conducted after Institutional Review Board approval. Informed consent was waived as there were no risks to patients.

We reviewed the medical records of our cancer patients, age >12 years, who had indwelling LCVCs for at least 2 weeks. By searching the infection control surveillance database and the microbiology laboratory database at our institution, we identified 200 patients who had their CVC inserted between January 2017 and April 2022 and developed a CRBSI during the study period. These cases were compared to 400 control subjects who were randomly selected through simple random sampling from a pool of 5,537 cancer patients who had a CVC, inserted between 09/2018 and 08/2022, in place for ≥two weeks and who didn’t have any documented CRBSI during that time. These patients were identified using electronic health records supported by the Epic system.

Data collected included patient demographics, clinical characteristics, CVC details, and time to CRBSI occurrence.

Chi-square or Fisher’s exact tests were used to compare categorical variables, and Wilcoxon rank-sum tests for continuous variables. Logistic regression analysis was used to identify independent predictors of CRBSI. First, univariate analysis was performed for the factors chosen based on clinical relevance and prior literature. Then variables with *P*-values < 0.15 from univariate analyses were considered for multivariate analysis and backward elimination procedure was used to reduce the full model to the final model in which only the significant factors (*P* < 0.05) remained. All tests were two-sided with a significance level of 0.05. The statistical analyses were performed using SAS version 9.4 (SAS Institute Inc., Cary, NC, USA).

### Definitions

CRBSI was defined according to the IDSA guidelines^1^ as bloodstream infection meeting one of the three criteria: (1) paired quantitative blood cultures: CVC colony count ≥3-fold higher than peripheral blood, (2) differential time to positivity (DTP): CVC culture positive ≥2 hours before peripheral culture, (3) matching organism in blood culture and catheter tip culture (≥15 CFU in semiquantitative or >10^2^ CFU in quantitative cultures).

## Results

Baseline characteristics were similar between CRBSI patients and controls (Table 1). Most catheters were placed on the right side of the body (67% in CRBSI vs. 71% in non-CRBSI; *P* = 0.21). However, patients with CRBSI were significantly younger (*P* < 0.001), had higher rates of hematological malignancies (*P* < 0.0001) and BMI ≥30 kg/m^2^, and were more likely to have experienced allogenic transplant, mucositis, GVHD, neutropenia, and thrombocytopenia (P < 0.001 for all).

**Table 1.** Baseline characteristics of patients with and without catheter-related bloodstream infection (CRBSI)

Characteristics^a^	CRBSI (n = 200)	non-CRBSI (n = 400)	*P*-value
Age, median (range), y	57 (11–85)	62 (11–89)	<0.001
Sex, male	111 (56)	201/298 (51)	0.25
BMI, kg/m^2^			0.015
<25	84 (42)	153/398 (38)	
25 to <30	46 (23)	136/398 (34)	
≥30	70 (35)	109/398 (27)	
Type of cancer			<0.0001
Hematological	164 (82)	172 (43)	
Solid tumor	36 (18)	226 (57)	
Both	0 (0)	2 (0.5)	
Diabetes mellitus	43/199 (22)	64/394 (16)	0.11
Hypertension	92 (46)	178 (45)	0.73
Chronic cardiac disease	16 (8)	86/399 (22)	<0.0001
Cirrhosis	0 (0)	5 (1)	0.18
Hemodialysis	5 (3)	5 (1)	0.31
Corticosteroid therapy^b^	35 (18)	55/394 (14)	0.26
Hypoalbuminemia	77/196 (39)	119/387 (31)	0.039
TPN	18/197 (9)	24/398 (6)	0.16
Transplant	71/199 (36)	68/396 (17)	<0.0001
Type of transplant			<0.0001
Autologous	11/71 (15)	46/68 (68)	
Allogenic	60/71 (85)	22/68 (32)	
Allogenic transplant	60/199 (30)	22/396 (6)	<0.0001
Neutropenic (ANC <500 cells/μL)	120/199 (60)	73/385 (19)	<0.0001
Thrombocytopenia	160/199 (80)	165/388 (43)	<0.0001
ICU admission	9 (5)	29/393 (7)	0.18
Mucositis	44/195 (23)	36/392 (9)	<0.0001
GVHD	17 (9)	9/398 (2)	0.0004
Permanent foreign body	27/199 (14)	113/398 (28)	<0.0001
Received blood products through the catheter	154/190 (81)	176/392 (45)	<0.0001
Known MRSA colonization	1/57 (2)	4/335 (1)	0.55
Known VRE colonization	10/140 (7)	6/348 (2)	0.004
Exposure to antibiotics^c^	170 (85)	200 (50)	<0.0001
Type of catheter			
Tunneled catheter or totally implantable port	13/199 (7)	139/399 (35)	<0.0001
Non-tunneled catheter	72/199 (36)	91/399 (23)	0.0005
PICC	114/199 (57)	168/399 (42)	0.0005
Number of catheter lumens			<0.0001
1	15 (8)	163/397 (41)	
2	135 (68)	202/397 (51)	
3	50 (25)	32/397 (8)	
Catheter site			
Subclavian	55 (28)	70 (18)	0.005
Jugular	17 (9)	115 (29)	<0.0001
Femoral	3 (2)	5 (1)	>0.99
Cephalic	7 (4)	2 (0.5)	0.008
Basilic	80 (40)	109 (27)	0.002
Brachial	27 (14)	59 (15)	0.68
Catheter removal	187/199 (94)	348 (87)	0.009
Length of catheter stay, median (IQR), days	73 (29–169)	56 (27–170)	0.57
Duration from catheter insertion to CRBSI, median (IQR), days	51 (23–136)	NA	

*Abbreviations*: ANC, absolute neutrophil count; BMI, body mass index; CRBSI, catheter-related bloodstream infection; GVHD, graft versus host disease; ICU, intensive care unit; IQR, interquartile range; MRSA, methicillin-resistant *Staphylococcus aureus*; NA, not applicable; PICC, peripherally inserted central catheter; TPN, total parenteral nutrition; VRE, vancomycin-resistant enterococci.

a Data are no. (%) unless otherwise indicated.

b Cumulative 300 mg prednisone equivalent.

c For 5 consecutive days or more, within 4 weeks prior.

Patients with CRBSI had a higher number of prior CVCs within the past year than did non-CRBSI patients (*P* < 0.0001) and were more likely to have catheters with multiple lumens (*P* < 0.0001). They were more likely to have non-tunneled catheters and peripherally inserted central catheters (PICCs) (93% vs. 65%, *P* < 0.0001). In CRBSI, the most common insertion sites were subclavian (28% vs 18%, 0 = 0.005) and basilic veins (40% vs 27%, *P* = 0.002), whereas in non-CRBSI it was jugular vein. Tunneled catheters or totally implantable ports were more common in the non-CRBSI group (7% vs. 35%, *P* < 0.0001).

Patients with CRBSI were more likely to have their catheters removed (94% vs 87%; *P* = 0.009). They were more likely to have received blood products, had a higher rate of vancomycin-resistant enterococci colonization, and prior antibiotic exposure (all *P* < 0.001).

CRBSI occurred after a median of 51 days from catheter insertion. The rate of CRBSI increased sharply from 2.5% at 2 weeks to 32.5% at 4 weeks, 53.5% at 8 weeks, 71.0% at 16 weeks, and 84.0% at 30 weeks.

Pathogens causing CRBSI included 62% gram-positive bacteria, 37% gram-negative bacteria, and 2% *Candida* species.

On multivariate logistic regression analysis, leading independent risk factors for CRBSI were allogenic transplants, neutropenia, multiple catheters within the past year, blood product administration, BMI >30 kg/m^2^, and PICC lines in the superficial basilic/cephalic veins. Jugular vein insertion was a protective factor against CRBSI (Table 2).

**Table 2.** Independent predictors of CRBSI by multivariate logistic regression analysis

Independent predictors	aOR	95% CI	*P*-value
BMI ≥30 kg/m^2^	1.75	1.10–2.77	0.017
Type of cancer			-*
Hematological			
Solid tumor			
Both			
Diabetes mellitus			-*
Hypoalbuminemia			-*
Allogenic transplant	3.36	1.83–6.17	<0.0001
Neutropenia (ANC <500 cells/μL)	3.06	1.95–4.79	<0.0001
Thrombocytopenia			-*
Mucositis			-*
GVHD			-*
Permanent foreign body			-*
Prior catheter(s) within the past year	2.79	1.78–4.36	<0.0001
Tunneled catheter or totally implantable port			-*
Non-tunneled catheter			-*
PICC			-*
Number of catheter lumens			-*
1			
2			
3			
Catheter site: jugular	0.41	0.21–-0.80	0.009
Catheter site: cephalic or basilic	1.70	1.07–2.71	0.024
Received blood products through the catheter	2.57	1.59-4.16	0.0001
Exposure to antibiotics			–*

*Abbreviations*: aOR, adjusted odds ratio; BMI, body mass index; CI, confidence interval, CRBSI, catheter-related bloodstream infection.

Note. * indicates that the variable was entered into the initial multivariate logistic regression model on the basis of the *P*-value on univariate analysis (≤ 0.15) and later removed from the final model through the backward elimination procedure.

## Discussion

This study identifies leading risk factors for CRBSI in cancer patients including allogenic transplant, neutropenia, multiple catheters, blood product administration, BMI >30 kg/m^2^, and PICC lines inserted in superficial basilic/cephalic vein, while jugular insertion was protective. Notably, 32.5% and 71.0% of all CRBSIs occurred at 4 and 16 weeks, respectively, following CVC insertion. Our findings have significant clinical implications for the management and prevention of CRBSI in high-risk cancer patients.

A unique feature of this current study is the use of the strict IDSA definition of CRBSI, to determine its risk factors in patients with LCVC.^1^ Unlike previous studies that relied on the broader CLABSI definition,^2^ which often overattributes infections in cancer patients with hematologic malignancies or transplants to vascular catheters rather than the gut where it mostly originates from, our approach, using the strict definitions, allows us to identify risk factors for true CRBSI.

Our study showed a rapid increase in CRBSI cases following LCVC insertion, from 2% at 2 weeks to 32.5% at 8 weeks and 71% at 16 weeks. A meta-analysis study showed that catheterization beyond 2 weeks was an independent risk factor for CLABSI in ICU patients.^3^ This rapid increase in CRBSI reflects the natural progression from catheter colonization to CRBSI. Safdar and Maki^4^ demonstrated that short-term catheters colonization is extraluminal, reduced by chlorhexidine application at insertion site, while LCVC infections originate from the catheter hub and lumen, with increasing risk from frequent catheter hub interceptions and excessive blood products administration. Previous studies showed that lock solutions and antimicrobial catheters could prevent intraluminal colonization and CRBSI in patients with LCVC.^5,6^

Our univariate analysis showed a significantly higher proportion of hematological malignancies among CRBSI patients, highlighting their vulnerability. This aligns with previous studies, which attributed the high incidence of CLABSI in these patients to prolonged neutropenia, reinforcing neutropenia as a key independent risk factor.^7^ We also identified prior allogenic transplant as a major independent risk factor for CRBSI which is consistent with previous studies.

Even though biofilm formation occurs nearly universally on CVCs, irrespective of blood product exposure,^8^ our multivariate analysis showed that receiving blood products through the CVC was a significant risk factor for CRBSI, consistent with studies linking transfusions to increased CRBSI risk.^9^ Blood products may promote biofilm formation on catheter surfaces by providing essential metallic ions like iron, calcium, and magnesium.

Having a prior LCVC within the past year was a significant independent risk factor for CRBSI, aligning with findings from a previous systematic review. In our study, patients with CRBSI had a higher proportion of non-tunneled catheters, likely due to clinical conditions such as hematological malignancies resulting in thrombocytopenia, which may preclude tunneled catheter placement, potentially contributing to a higher CRBSI risk in non-tunneled catheter.

Furthermore, our study found that a BMI of 30 kg/m^2^ or higher was an independent risk factor for CRBSI which is consistent with other studies.^10^

Finally, our study is unique in demonstrating that PICC lines inserted in the superficial basilic/cephalic vein carry the highest CRBSI risk among LCVC sites, while those in the deep brachial vein were not associated with CRBSI. Previous studies showed that patients with PICC in basilic/cephalic veins were more likely to develop upper extremity venous thrombosis.^11^ Other studies demonstrated that thrombotic complications of LCVC were associated with catheter-related bacteremia. Thus, PICC lines placed in the superficial basilic/cephalic veins (as opposed to the deep brachial veins) are associated with higher rates of venous thrombosis, predisposing patients to a higher risk of CRBSI. Jugular vein insertion of a CVC was protective against CRBSI.

This study’s major limitation is its retrospective design, which precludes establishing causality between identified risk factors and CRBSI development. The single-institution study population limits generalizability, as do potential confounders like variations in catheter care protocols. Lastly, our CRBSI definition may be overly specific while the CLABSI definition may be overly broad.

In conclusion, key risk factors for CRBSI in cancer patients with LCVCs include allogenic transplant, neutropenia, blood products administration, prior LCVC within 1 year, BMI ≥30 kg/m^2^, and PICC in superficial basilic/cephalic veins. In contrast, internal jugular vein insertion was associated with lower CRBSI risk. Preventive interventions such as insertion of PICC in deep rather than superficial veins, antimicrobial locks, and antimicrobial catheters with prolonged activity beyond 2 weeks are warranted in the highest-risk populations.
